# Robustness against Chirp Signal Interference of On-Board Vehicle Geodetic and Low-Cost GNSS Receivers

**DOI:** 10.3390/s21165257

**Published:** 2021-08-04

**Authors:** Franc Dimc, Polona Pavlovčič-Prešeren, Matej Bažec

**Affiliations:** 1 Faculty of Maritime Studies and Transport, University of Ljubljana, Pot Pomorščakov 4, SI-6320 Portorož, Slovenia; matej.bazec@fpp.uni-lj.si; 2 Faculty of Civil and Geodetic Engineering, University of Ljubljana, Jamova Cesta 2, SI-1000 Ljubljana, Slovenia; polona.pavlovcic-preseren@fgg.uni-lj.si

**Keywords:** precise GNSS position determination, carrier-phase positioning, interference mitigation

## Abstract

Robust autonomous driving, as long as it relies on satellite-based positioning, requires carrier-phase-based algorithms, among other types of data sources, to obtain precise and true positions, which is also primarily true for the use of GNSS geodetic receivers, but also increasingly true for mass-market devices. The experiment was conducted under line-of-sight conditions on a straight road during a period of no traffic. The receivers were positioned on the roof of a car travelling at low speed in the presence of a static jammer, while kinematic relative positioning was performed with the static reference base receiver. Interference mitigation techniques in the GNSS receivers used, which were unknown to the authors, were compared using (a) the observed carrier-to-noise power spectral density ratio as an indication of the receivers’ ability to improve signal quality, and (b) the post-processed position solutions based on RINEX-formatted data. The observed carrier-to-noise density generally exerts the expected dependencies and leaves space for comparisons of applied processing abilities in the receivers, while conclusions on the output data results comparison are limited due to the non-synchronized clocks of the receivers. According to our current and previous results, none of the GNSS receivers used in the experiments employs an effective type of complete mitigation technique adapted to the chirp jammer.

## 1. Introduction

Any source that interacts with GNSS signals interferes with the process of estimating the propagation time. The approach of spreading the spectrum of the GNSS signal, optimised antenna and spatial, time and frequency domain filter designs allow a large number of interfering signals to be mitigated [1,2]. In this research, the authors investigate how successful these mitigation techniques are in cases where moving GNSS receivers are used at the output data level, involving a set of spatially well-defined receivers in the network of static reference stations. Autonomous vehicles should georeference with reliable and accurate GNSS receivers; consequently evaluating the quality of positioning in kinematic mode within the in-L1-band interference signal environment is of interest to many researchers [3,4,5,6,7]. The authors’ objective was to optimise the GNSS hybridisation with three professional-grade geodetic receivers and additionally three low-cost navigation GNSS receivers, all of them passing by a static jamming source. In particular, the authors were also interested in the sensitivity of the positioning effect to the rather small change in the relative height of the receiver antenna to the interference source.

Thanks to applied mitigation techniques, modern GNSS receivers become insensitive to interference from poorly designed communication systems or interference from malfunctioning electronic equipment in daily use. Software (cognitive) radio solutions equip all processing chains in such a way that the receiver is able to detect and also mitigate the interference [8]. In this context, an essential question arises: “Do the applied mitigation techniques in the different branded carrier phase tracking devices introduce a significant bias to pseudoranges, and, as in the case of the geodetic receivers, do they affect the carrier phase and thus degrade the kinematic positioning approach?” To answer the question experimentally, and without the knowledge of implemented software solutions, the authors exposed several GNSS devices to the same interference signal at the same satellite constellation and also experimented with more combinations of them.

### 1.1. Overview

In the wide range of GNSS receiver applications, from professional and safety-of-life preserving to social and recreational applications supporting location-based services, geodetic receivers are generally not a target of intentional interference. However, carrier-phase-based positioning techniques used for geodetic surveys are very likely to be a victim of adversaries when such techniques support high-integrity autonomous driving, flying or sailing, especially in dense traffic situations. Loss of lock in a vehicle receiver’s carrier tracking loop at a crucial moment, for example, can result in at least one safety-related incident.

The goal of interference mitigation techniques is to ensure the most accurate and truthful solution by reconstructing the genuine signals and, consequently, their content. Open field campaigns, usually preceded by simulations, lead to relevant and rational approaches being found to mitigate the impact of real attacks on GNSS receivers from various sources with different types of signals [9]. Manufacturers of receivers have put great effort into research to overcome the problems of intentional degradation, with great success. Although they have their own specific anti-jamming solutions, their antennas all adopt rejection of low elevated satellite signals and receiver signal processing-based techniques, also enhanced by null steering techniques. On the side of receiver, there are several pre-correlation techniques for the mitigation of interference in the receiver that try to eliminate high frequency interference on measurements prior to the correlation process. Mitigation techniques solve either affected front-end, acquisition or tracking stage and estimate their success by improving the initial signal-to-noise ratio. Analogous to the sparseness of the interference signal in a particular domain, there are already implemented techniques to overcome either short interference pulses, namely pulse blanking [10,11], or the spectral narrowband interference suppression technique called notch filtering [12], which also diminishes the influence of continuous waves interferences [13]. By using filters to reduce interference, receivers can introduce bias into measurements. Fixed-notch devices, for example, may introduce biases that can be estimated and compensated for [14,15,16,17]. Adaptive notch filters are an effective solution for mitigating jamming [18], which are able to track the frequency variations of a jamming signal, pulse suppression or adaptive beamforming based on the multi-antenna solution [19,20]. To improve the robustness of the code and carrier tracking loop, Susi and Borio proposed a single adaptive Kalman filter, fed by non-coherently integrated discriminator outputs that improve the dynamic performance of data processing when tracking moderately degraded Galieo E6-B signals in the presence of multipath and fading [21]. For similar interference conditions, based on a simulated static and dynamic scenario when tracking a single GPS satellite signal, a joint group from Fraunhofer Institute for Integrated Circuits and Tampere University [22] proposed an adaptive phase-locked loop bandwidth technique, namely LBCA, with piecewise linear approximation of nonlinearities.

Deviations from normal GNSS signals-in-space conditions caused by interference at the receiver stage could be detected by monitoring the behaviour of GNSS signal processing blocks, starting with the automatic gain control and ending with the position, velocity and time (PVT) solution block [9]. The intrinsic idea of the project known as “Standardisation of GNSS threat reporting and receiver testing through international knowledge exchange, experimentation and exploitation (STRIKE3)”, which was active under the European Global Navigation Satellite Systems Agency from 2016 to 2019, was to set up L1 band interference monitoring systems in 23 European countries [23]. From a total of 450,000 interference events over the two-year period, 66,000 were determined to be jammers [24,25]. Jamming is usually used to prevent a vehicle being tracked through GNSS; however, when used a jammer also impacts other devices that are used for positioning and timing services, including continuously operating reference station infrastructure, which is used for geodetic purposes or autonomous navigation. Earlier studies at the University of Ljubljana showed that none of the professional geodetic and low-cost GNSS receivers used in the experiments had an effective type of full jamming suppression adapted to the chirp jammer, but receivers of newer generations showed a dramatic improvement against jamming [26,27]. This was particularly true for the GLONASS satellite signals, but in some cases also for Galileo and BeiDou, which were found to be less susceptible to interference from the L1 chirp jammer. Moreover, an interesting property has been recognised that applies to geodetic and low-cost GNSS receivers, i.e., that it is more difficult for simple jammers to jam GNSS receivers installed higher than the jammer’s usage horizon [26]. This fact is important because in most cases, GNSS continuous operating stations are located in urban centres higher than street level, where simple jammers are usually deployed. Wendel et al. [28] report that several interference-monitoring systems have been developed by the European Space Agency (ESA) with the goal of protecting ranging and integrity-monitoring stations (RIMSs). A commercial off-the-shelf signal analyser has been developed that uses sophisticated detection algorithms and is used to monitor the 800–1800 MHz frequency bands. When jamming is detected, it should be analysed through the electromagnetic spectral environment to determine the effect on positioning and time transfer, and the interference signal should also be classified. With such an approach, unauthorised transmitters can be detected or even located in good scenarios.

The goal of conducting real-life interference experiments, if possible, is to gain more insight into the GNSS measurements affected by interference and the final processing product, i.e., positions, which is archived for possible offline analysis. From the tagging, the time and duration of occurrence can be determined. The extracted information can be used to compare future characterised features for possible detection. This information can then be used to provide insights into the source of interference as well as a means to remove interference in any GNSS equipment used for time transfer, high-quality positioning or navigation. The aim is to avoid, or at least detect, interference through the use of any GNSS equipment, and possibly to report it, especially near critical infrastructure that relies on GNSS and in high density traffic, such as near airports, ports or roads.

### 1.2. Paper Focus and Outline

In the context of this state-of-the-art overview, the driving motivation for the research presented in this paper is to conduct further experiments, starting with detecting and locating jammers [29], and especially, as a follow-up to the 2019 and 2020 experiments [26,27], by using static jamming of receivers that are in kinematic mode, and to analyse the results so that a better understanding of some of the main factors that influence signal reception during jamming can be obtained. The research is based on the results of previous studies conducted at the University of Ljubljana, which showed that the response of receivers from different manufacturers and generations is unique for each type of receiver tested; however, the newer generation of professional geodetic receivers is much more resistant to jamming [26,27].

The basic research question addressed is: “Is there a significant difference in the reception of satellite signals from different GNSS receivers placed on a vehicle and in kinematic mode during jamming?” More specifically, this research focuses on two different aspects:

- The effect of static jamming, where a jammer is placed at different heights and a vehicle with receivers passes the jammer at a constant speed;
- The quality of signal reception and the re-acquisition time after jamming for different receivers.

The added value of this study and the improvement over previous studies by the authors is the analysis of the quality of positioning during jamming for vehicles equipped with GNSS passing the interference source. This is particularly important for the direct georeferencing of unmanned aerial vehicles (UAVs) in real time and evaluating the quality of georeferencing in the presence of GNSS signal jamming. The innovation of this study lies firstly in the extended quality representation of the behaviour of various receivers, including their clocks, operating in kinematic mode in the presence of jamming. Secondly, the great significance of the experiment lies in the comparison of the behaviour between receivers of different quality, i.e., high-quality and reliable geodetic receivers and low-cost but affordable GNSS receivers that have recently appeared on the market. This is mainly due to the fact that low-cost receivers will be widely used for real-time kinematic navigation, as their usability in difficult conditions due to damage or destruction is not as risky as the use of professional and very expensive geodetic receivers.

The results of these experiments are presented numerically and graphically, and a discussion of the analysis and the authors’ interpretation of the results are given for each part of the research. The paper is based on the following structure: Section 2 describes the testing site and measurement campaign including the receivers used in the experiment; Section 3 evaluates the results from the experiments and continues with the discussion; Section 4 contains conclusions.

## 2. Materials and Methods

### 2.1. Setup and Measurement Campaign

The test site was established in July 2015 near the village of Črnotiče in Slovenia, which has previously been used for several experiments [26,27,29]. The site is located in a remote area with almost no traffic, so the impact of interference on users is minimal. In addition, the road is straight from northwest to southeast and without any bends for a length of about 1.5 km. There were almost no obstacles on the part of the road where the experiments took place that would disturb the GNSS signal reception. If there had been any obstructions, they would have been at the more distant points of the road in relation to the position of the jammer. There was almost no traffic, and the straight road made it possible for the vehicle to be driven at a constant speed. The reason for choosing the test area within an almost straight road was also that the orientation of the antennas remained fixed all the time and was not corrected during the experiment. In this way, the influence of different antenna orientations could be considered equally for all positions within the vehicle trajectories. The most important point in relation to the fact that the use of a jammer is illegal and prohibited by law is that the use of a jammer was approved by the Agency for Communication Networks and Services of the Republic of Slovenia (AKOS).

**Figure 1:**
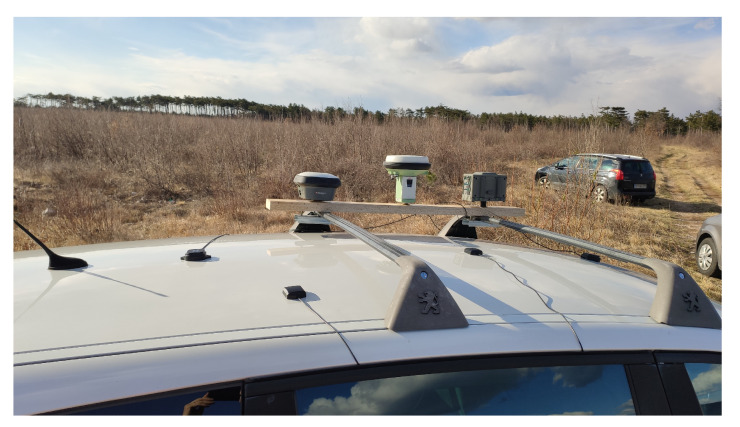
GNSS receivers set up on the roof of the vehicle (own study).

The experiments were conducted on the 78th day of 2021 (19 March 2021). The observations were performed from about 13:45 to 16:30 UTC with a jamming time of 14:00 to 15:00 UTC. Receivers from various manufacturers were used in the experiment, namely: Javad GNSS Inc. (San Jose, CA, USA), Leica Geosystems AG (Heerbrugg, Switzerland), ArduSimple (Lleida, Spain) and U-blox (Thalwil, Switzerland). Three GNSS receivers, namely Leica GS18T (antenna type: LEIGS18 NONE), were placed at the most distant locations, as they played the role of a base receiver in the kinematic GNSS positioning. Three geodetic instruments, Javad Triumph-LSA (antenna type: JAVTRIUMPH_LSA NONE) with internal antennas, Leica GS15 (antenna type: LEIGS15 NONE) and Leica GS18T (antenna type: LEIGS18 NONE), and three U-blox modules, namely ZED-F9P and ZED-F9R with ANN-MB00 multi-band GNSS antennas [30,31] and the U-blox M8T module were placed on the roof of the vehicle (Figure 1). From those, only the Leica GS15 receiver and the U-blox M8T could not receive signals from BeiDou.

**Figure 2:**
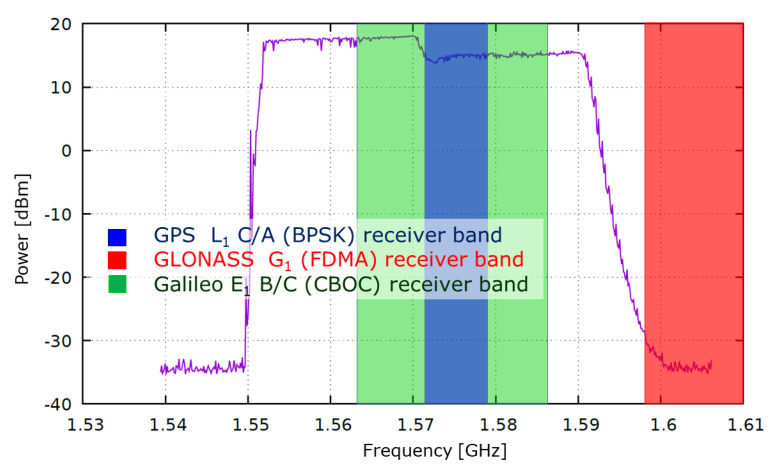
Peak hold power spectrum of the jammer’s output compared with typical front-end bandwidths of GNSS receivers (own study).

As in the previous two experiments [26,27], a commercially available unmarked L1/E1 jammer with sub-miniature (SMA) connector was used for the experiments. The external omnidirectional antenna of the jammer emits a single sawtooth chirp signal, and belongs to class II. The jammer’s external antenna with an omnidirectional radiation pattern was connected through an SMA connector, which emits a single saw-tooth chirp signal and belongs to class II. The output of the jammer has a period of 10 μs [32,33,34]. In terms of the test conducted for this study, the device signal output increased the noise power, as presented in Figure 2, by up to 50 dB in a frequency band of 1570±20 MHz.

The signal of the jammer changes its spectral characteristics over time in the form of a frequency modulated narrowband signal and thus interferes with a wider spectrum. Following the STRIKE3 experiment for standardised threat reporting of jamming events [23], the effect of this particular jammer can be described as a type B jamming event, which causes a 10 dB decrease in CNR that lasts longer than 5 s [35]. The jammer was located close to the road; its horizontal position remained the same, but vertically the height was changed between 157, 213 and 268 cm above the ground.

**Figure 3:**
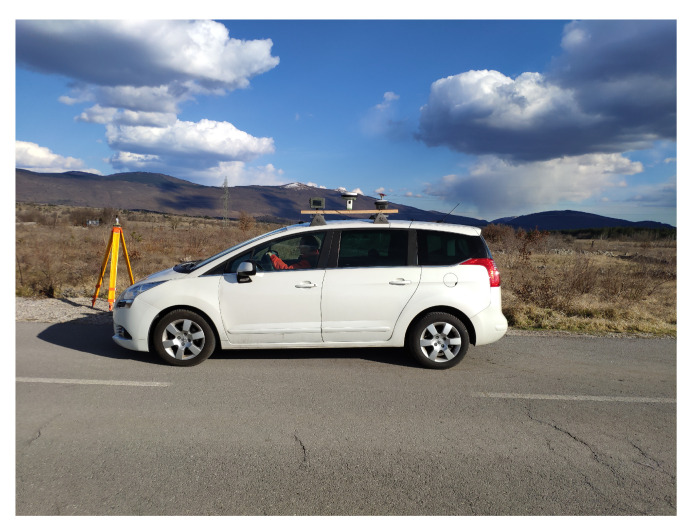
Vehicle with the GNSS receivers on the roof passing by the road static jammer (own study).

**Figure 4:**
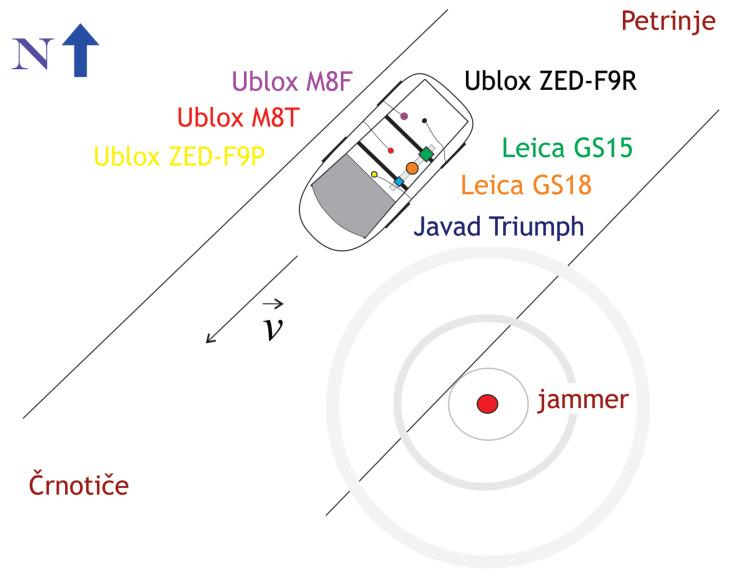
The principle of localising of the moving vehicle with an array of GNSS receivers, while the jammer is set statically by the road (own study).

Jamming for the moving vehicle equipped with GNSS receivers was then performed using a jammer located by the road (Figure 3), where the vehicle moved back and forth between Črnotiče (C) and Petrinje (P) by passing the jammer at location J (Figure 4, Table 1). In Table 1, the coordinates for the most distinct points were determined with the RTK method, and thus the accuracy is presented within centimeters. The start and end times for each successive driving are presented in Table 2.

**Figure 5:**
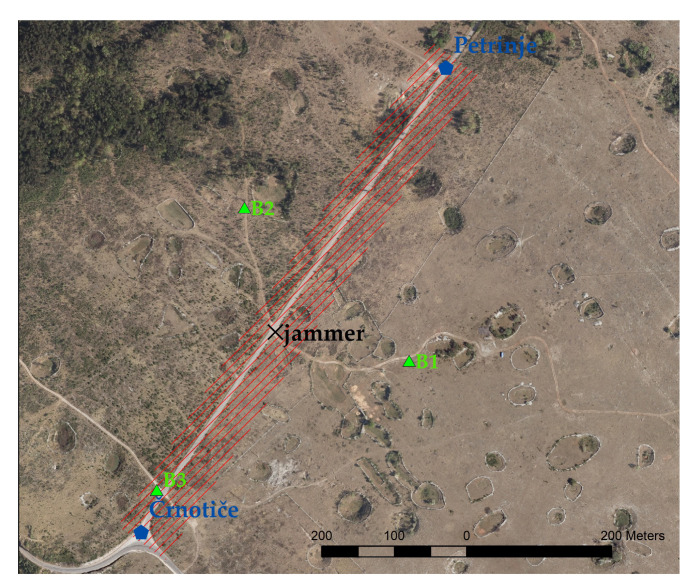
Positions of the base receivers (B1–B3), jammer and directions of movement of the vehicle (towards Petrinje and Črnotiče and vice versa) (own study).

Just in case the receivers might somehow suffer any intentional interference, three base receivers were set at three different locations (Table 3, Figure 5) for further kinematic pos-processing. Indeed, such an occurrence took place at site B1, when the jammer was 268 cm above the ground. Therefore, the base receiver of point B2 was used for further analysis in post-processing. The observation time rate was set to 0.1 s for all geodetic receivers, and 0.5 s for the three U-bloxes due to previous problems with registering observations at the 0.1 s time rate. Positions for three potential base receivers (eventually the receiver at point B2 was chosen) were determined by a static post-processing procedure using a virtual reference station (VRS) from the Slovenian CORS network SIGNAL. The local network consisted of redundant baselines between points B1–B3 and the VRS; therefore, the coordinates of points B1–B3 were determined with a GNSS network least-squares adjustment. The estimated accuracy was a few millimetres, and thus the coordinates in Table 3 are presented with better accuracy than those from Table 1.

For test drives 1 to 5, the jammer was positioned 157 cm above the ground, but at the same height as the receivers on the vehicle (the difference between the height of the jammer and the receiver was 9 cm). For test drives 6 to 9 the height was 213 cm above the ground and 0.65 m above the receivers on the vehicle’s roof. For the remaining test drives the height was set at 268 cm above the ground and 1.20 m above the receivers on the vehicle. The reason for placing the jammer on the receiver’s horizon or higher came back to an earlier experiment [26] where there was less obvious interference when the jammer was below the receiver’s horizon. During test drive 15, the driver accidentally stepped on the accelerator pedal causing the speed to fluctuate on the track section. Only for this drive, the velocity was about 80 km/h.

### 2.2. Observation Processing

Since relative GNSS processing based on differentiation of carrier-phase observations effectively eliminates orbital and atmospheric influences, it is also widely used in kinematic positioning. To determine the positions at a high-quality level, carrier-phase ambiguities should be resolved as integers (fixed values). To determine the positions of the receivers placed on the roof of the vehicle, positioning was performed with the base receiver placed near the test area but far enough away so that interference did not affect the reception of signals from the satellites. The reason for not using the products of the Slovenian continuously operating reference station network SIGNAL was the fact that the receivers on SIGNAL were able to receive observations from GPS, GLONASS and Galileo, while the base receivers used in this experiment could also receive BeiDou. However, the positions of the B1-B3 base receivers (Table 3) were determined by using SIGNAL.

In the kinematic experiments, the time rate for signal reception was set to 0.1 s for the geodetic receivers, while it was set to 0.5 s for all three U-bloxes. The custom software receiver was used to generate the Receiver Independent EXchange format (RINEX) from binary files; however, for some receivers, such as Leica GS15 and Leica GS18T, RINEX was already generated in the receiver. RINEX files from each of the receivers tested were used for an analysis of the quality of the measurements and position. Decision for testing receivers in post-processing kinematics (PPK) rather than real-time (RTK) arose from the fact that geodetic receivers were able to acquire coordinates for the 1 s sampling rate in RTK, but in this study a higher sampling rate of 0.1 s was under investigation and was achieved using PPK. However, prior to this experiment, another study was conducted to compare the RTK and PPK coordinates for the 1 s sampling rate where a consistency of 2 cm was achieved between the two methods.

The GNSS observations were processed using the RTKLIB software (demo5_b33e) [36,37], using all of the available navigation constellations and broadcast ephemerides. Since the post-processing, a combined mode with continuous ambiguity resolution was used instead of the forward mode with activated fix-and-hold. Coordinates were acquired for the various processing approaches, namely:

- Separately for each satellite system constellation, namely GPS, GLONASS, Galileo and BeiDou;
- For different combinations of constellations, namely GPS+GLONASS, GPS+Galileo, etc.

The approach to the evaluation of jamming kinematic vehicles followed three aspects; first, the determination of the sudden changes in carrier-to-noise power spectral density ratios for each satellite of each GNSS constellation; second, the possibility of position determination in relative kinematic processing for the fixed mode; and, finally, determination of the velocity for each receiver used in the experiment based only on quality defined positions. Despite the fact that the primary goal of the experiments was not to determine the velocities of the vehicle but the positions on the trajectory and thus to connect the influences of the environment with the positions, Doppler measurements were used in the post-processing to determine the velocity vector components. In addition, the quality of the clock operation at the time of GNSS jamming was assessed by post-processing for each receiver separately.

### 2.3. Jamming Detection and Quality of Mitigation

Among all the measurements that GNSS receivers provide, they may all suffer from the presence of jamming. Interference detection can be implemented by exploiting carrier-to-noise density ($C/N_{0}$, also known as CNR) measurements [38,39], as the presence of a jammer can be suspected by a significant drop in the CNR values [40,41], resulting in code and/or phase observations not being recorded. The attenuation of the jamming quality can be observed indirectly by comparing the responses of devices of different quality to jamming [26,27]. According to the fact that the antenna is the first line of defence against high frequency interference, and that mass-market GNSS receivers use antennae with little or almost no suppression, while on the other hand professional GNSS receivers are equipped with antenna arrays to dynamically change the antenna gain pattern [19,20,42], the quality of interference mitigation of mass market receivers was investigated in relation to professional GNSS receivers.

### 2.4. Position Determination

Due to the presence of the jammer, it was impossible to determine a precise position through direct measurement. Instead, it was decided that a computational approach should be used. From the conclusions of the authors’ previous research [26,27] and from a superficial inspection of the results, a decision was made to use the results from Leica GS18T (as the most reliable of the instruments involved) as a base for further processing. It should also be mentioned that alternative non-GNSS-based geodetic techniques, such as tachymetric measurements, were not an option due to the relatively fast car motion and high sampling rate.

It should be stressed from the outset that such an approach is only possible for relatively straight and uniform motion, where only minor deviations from the latter are expected. For this purpose, only a section of measurements was used where the velocity had already been stabilised and no sharp turns were involved. Some test drives were also excluded from the analysis due to the lack of uniformity.

First, the velocity was smoothed. Instead of only involving adjacent measurements in its calculation, it was calculated using a broad time interval of 2 s:

(1) $$\bar{u_{x}}\left(t\right)=\frac{x(t+\Delta t)-x(t-\Delta t)}{2\Delta t},$$

where *x* denotes one of the directions (northing, easting and height), $u_{x}$ its speed and Δt = 1 s. It should be noted that such a definition is equivalent to calculating the average of velocities (calculated from adjacent positions) over the interval of 2Δt. The position was then determined through integration of the smoothed velocities. The integration constant was chosen such that the weighted least square function *R* was minimal:

(2) $$R=\sum_{i}a_{i}{\left(x_{i}-{\bar{x}}_{i}\right)}^{2},$$

where $x_{i}$ is the measured position, ${\bar{x}}_{i}$ is the running average and $a_{i}$ is the weight that is equal to 1 if the measurement is within a three standard deviations interval and 0 otherwise. It should be stressed that such a calculation should be performed iteratively, since the knowledge of ${\bar{x}}_{i}$ and $a_{i}$ requires *R* to be already minimal.

In normal circumstances, such an approach would produce nothing but a running average of the position over an interval of 2Δt. However, such an approach allows the reasonable determination of position, even in regions where measurements are lacking due to the presence of a jammer. This can be done via a simple interpolation of the velocity in the region under consideration.

**Figure 6:**
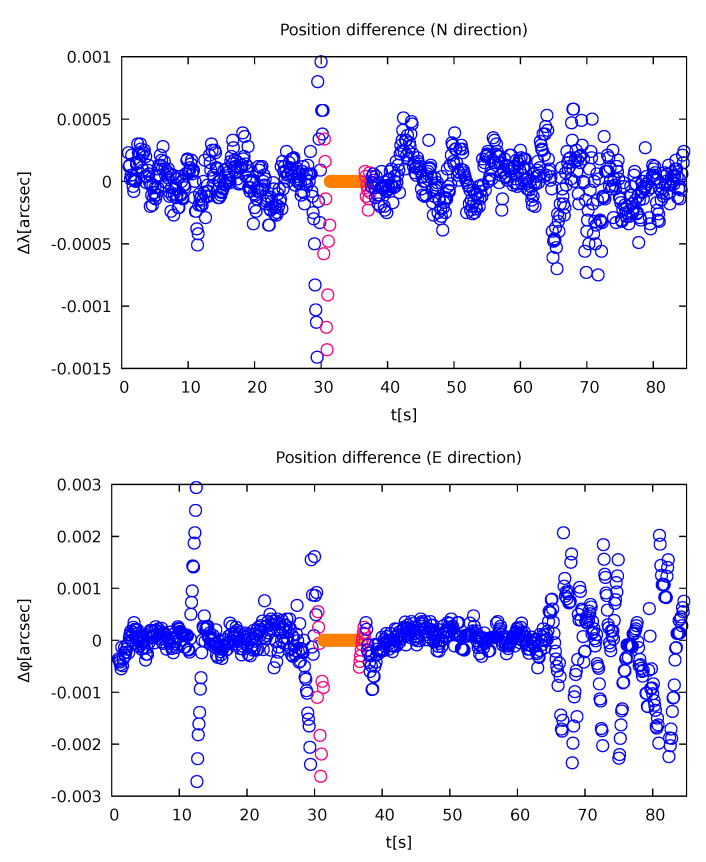
Variability of the resulted position differences. The blue points represent regular measurements; magenta shows the measurements that were still obtained but whose running average could not be otherwise calculated due to a point missing in their vicinity; the orange points represent the region where no measurements were taken—they were set to zero, since there is nothing to compare with and they were drawn for reference only (own study).

As can be seen in Figure 6, such an approach gives a reasonable determination of position, since it cancels out the fast fluctuations that are probably the consequence of a measurement artefact. For the sake of clarity, only one test drive is shown (test drive 2 from Table 2). The accuracy of the method can be estimated from those figures: below 1 cm in the regular regions and 5 cm in the jamming region safe for some screened regions. Note the increased fluctuation after 65 s and in a short interval around 10 s. These could be attributed to screening of the satellite signal due to the increased presence of the trees by the road. Furthermore, at 66.5 s the car drove over a communal shaft on the road that produced intensive vibrations that could possibly also affect the observables. Note also that the discrepancy is very much pronounced in the driving direction in comparison to the off-track direction.

**Figure 7:**
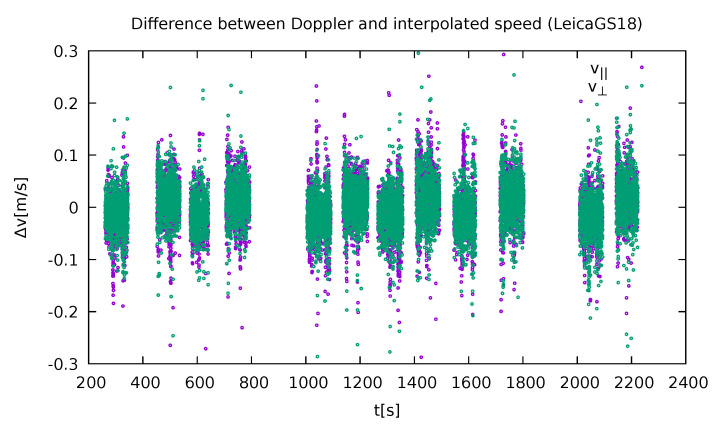
Smoothed velocity data compared to the measured GNSS Doppler measurements in the parallel and perpendicular directions with respect to the road (own study).

The method was also evaluated with respect to the GNSS Doppler measurements (Figure 7). The velocity components parallel and perpendicular to the road were compared. Both values were the same within a 0.1 m/s margin with only a few points varying more. The similarity also validates the position-determination method. There is, however, an interesting artefact that is beyond the scope of this paper that can be observed in the same figure. While both the components show almost the same behaviour, there is a small offset between the odd and even runs (corresponding to the two driving directions).

**Figure 8:**
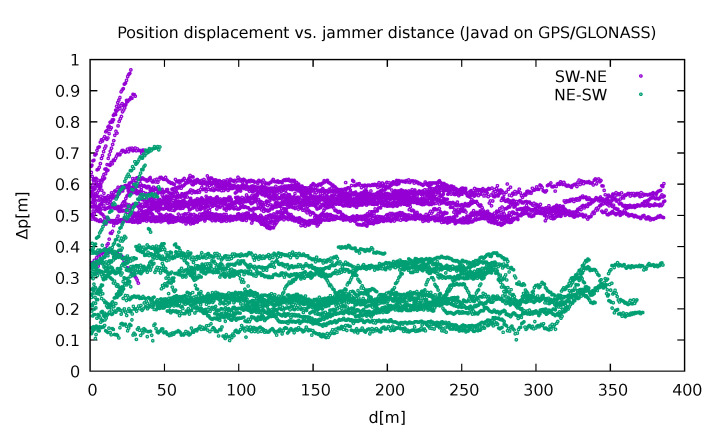
Discrepancy between the measured position and the calculated one for Javad Triumph LSA using GPS and GLONASS depending on the jammer distance (own study).

The position of other receivers (others than Leica GS18T) was supposed to be deduced from the same data set, by taking into consideration the relative position of the instrument with respect to the Leica GS18T, which was measured manually, and the orientation of the car. However, from Figure 8 it can be deduced that in the case of the Javad Triumph-LSA receiver using GPS and GLONASS, the position displacement fluctuated around different values for each direction.

**Figure 9:**
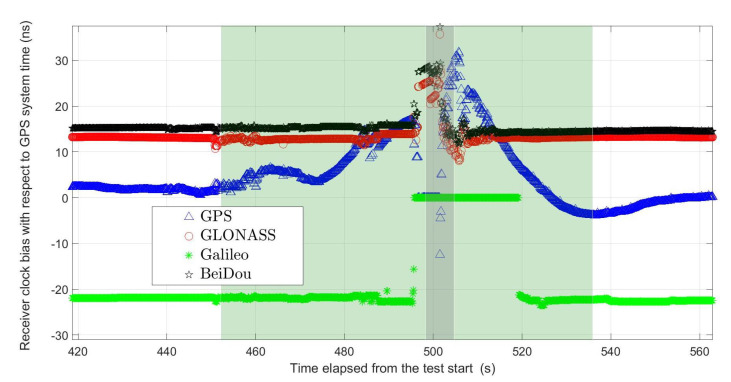
Clock biases for Leica GS18 during drive No. 2. The lighter shaded area represents the time interval of that drive, while the darker shaded area represents the interval when the antenna was closest to the jammer (own study).

There was some speculation at the beginning as to whether this might be the consequence of non-synchronised clocks. However, this possibility was excluded after some inspection of the instruments’ internal clocks and their comparison with the GNSS obtained time. From the typical behaviour of the clocks shown in the Figure 9 it is not only evident that the bias of the clocks did not exceed 50 ns, but also that a significant variation of the bias occurred the closer the receiver was to the jammer.

At the end it was deduced that such small time discrepancies (less than 1 μs, but usually much less) could not produce such substantial differences in position. This artefact is present in all the receivers and the authors cannot find any reasonable motivation for it. A proper understanding of this fact would probably require some insight into the algorithms used by the receivers. Unfortunately, this piece of information is virtually impossible to obtain from the manufacturers.

## 3. Results and Discussion

### 3.1. Reported Solution Quality

To start, the receivers were inspected for the ability to find an appropriate solution. For this purpose, the ratio of fixed solutions over the number of expected solutions according to the sampling rate was calculated. The same was evaluated for float solutions and joined to the fixed ratio in order to obtain a solution for any kind of ratio.

**Figure 10:**
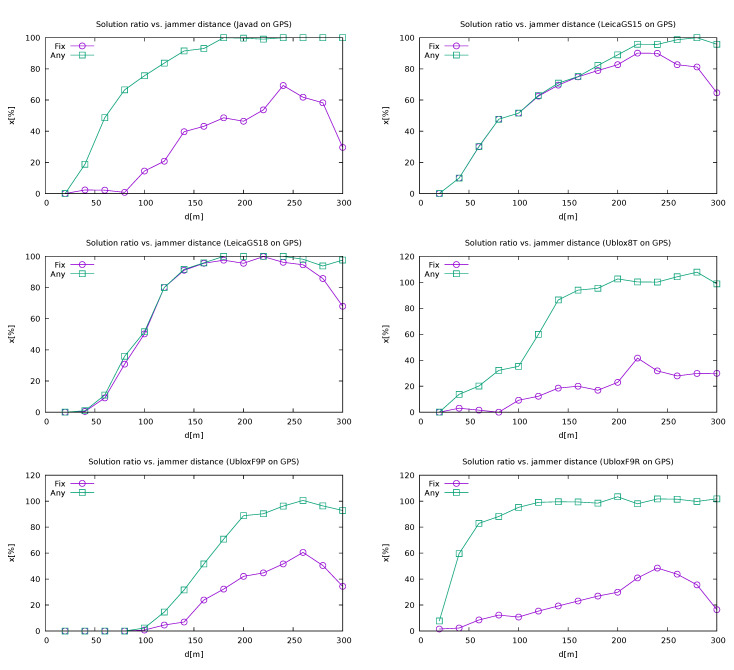
Quality reporting of different receivers depending on the jammer distance (non-cumulative) when using GPS only (own study, from the collection [43]).

The quality reporting provides a good criterion for the comparison of various receivers. For this reason, both ratios were calculated over a distinct distance from the jammer intervals. For the purposes of comparison, the positions were calculated using GPS only, since it is supported by all receivers and usually gives the best results. As can be seen in Figure 10, both the Leica receivers (GS15 and GS18T) outperformed the others, with GS18T performing slightly better. Among the other receivers, Javad Triumph-LSA performed better than the three U-blox-based receivers.

The ratios were calculated in the following way. The bins representing the distance from the jammer were divided into 20 m long intervals. Every 0.1 s the position of the instrument was known. This led to a total number of approximately 650 events per bin. Then the actual number of calculated solutions was counted, as well as the number of fixed solutions among them. From those values the ratios were calculated. The exceptions were made to all U-Blox receivers that had lower sampling ratios (0.5 and 1 s). For those, the result was normalised accordingly by a multiplication by 5 or 10. The normalisation step could lead to values slightly above 100%.

**Figure 11:**
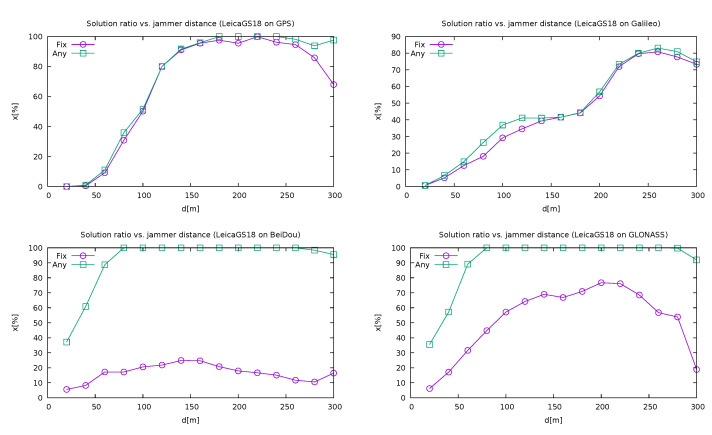
Quality reporting depending on the jammer distance (non-cumulative) on Leica GS18T when using different single satellite systems (own study, from the collection [43]).

Figure 11 shows how the Leica GS18T performed on different satellite systems, using a single system only for position determination: GPS, Galileo, BeiDou and GLONASS. As expected, GPS was the most suitable for position calculation, followed by Galileo and GLONASS, with BeiDou well behind. However, in the regions in the proximity of the jammer, GLONASS performed better than the others, which is not surprising, since it operates in a slightly higher frequency band that is outside the jamming region [32]. It is also interesting that in the case of GPS and Galileo, when a solution is present, it is almost always fixed, in contrast to GLONASS and even more to BeiDou. However, those results might not be very representative, since among the present GLONASS satellites there was only one on an elevation above 40 degrees at the time of the experiment.

### 3.2. Carrier-to-Noise Ratio

**Figure 12:**
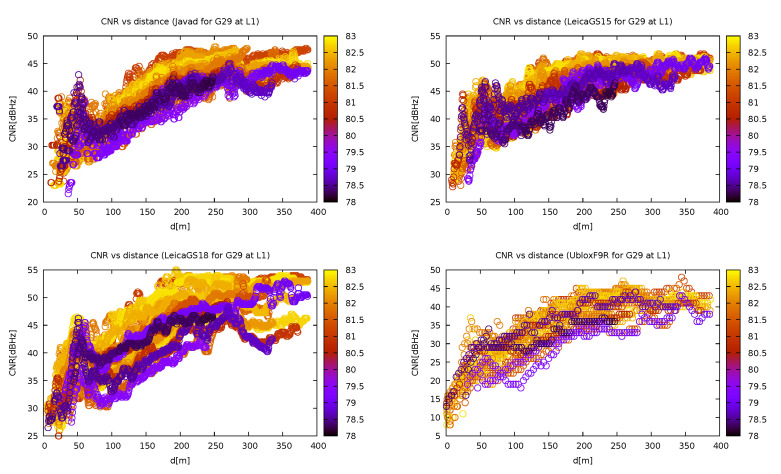
Carrier-to-noise ratio (CNR) of the GPS satellite G29 dependency on distance from jammer for various instruments. Different colours represent different elevations (own study, from the collection [43]).

The study of the dependence of the carrier-to-noise ratio showed that the receivers perform worse in jammer proximity, which was entirely expected (for instance, in the case of G29 in Figure 12). From the same figure, it can be deduced that the satellite reception of both the Leica instruments performed the best, followed by Javad Triumph-LSA and U-blox. The latter was also the most affected by the presence of the jammer. This can lead us to a conclusion that both Leica receivers bring out more signal from the background, probably due to better RF signal filtering. However, since no instrument performed significantly differently from the other (the shape of the CNR dependency did not vary much among them), it can be assumed that none uses any of the interference mitigation techniques adapted to this sort of jammer.

**Figure 13:**
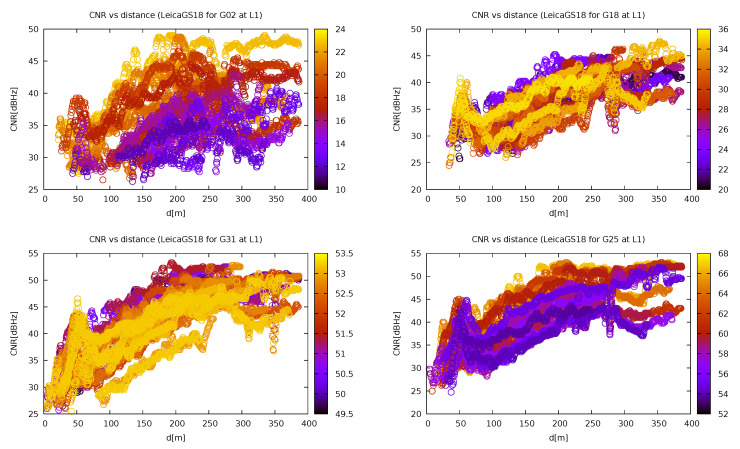
Carrier-to-noise ratio (CNR) of the GPS satellites with different elevations depending on the distance from the jammer for Leica GS18. Different colours represent different elevations (own study, from the collection [43]).

When inspecting the effect of the satellite elevation on the CNR on jammer distance dependency, it can be seen in Figure 13 that the dependency had the same shape in almost every case, except for the lowest elevations, where the reception is bad on its own even in non-jammed regions and thus the jammer does not have such an impact.

**Figure 14:**
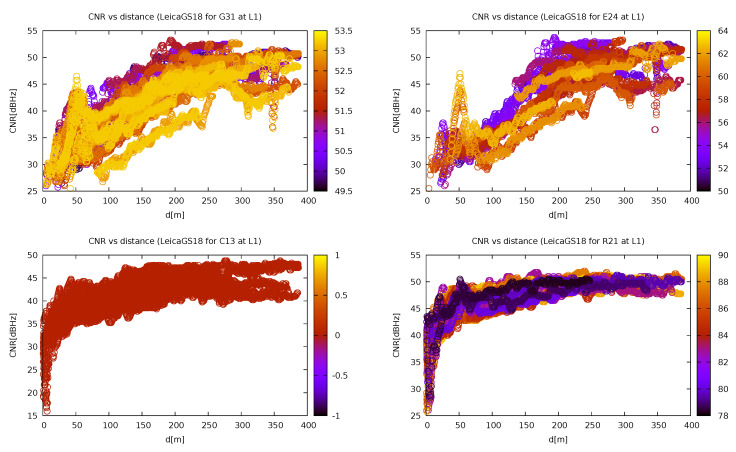
Carrier-to-noise ratio (CNR) of different GNSS satellites at similar elevations depending on the distance from the jammer for Leica GS18T. Note that the elevation for the BeiDou satellites was not reported correctly. Different colours represent different elevations (own study, from the collection [43]).

To end, the effect of the jammer on satellites of different GNSSs was investigated. For this purpose, a study of four satellites at approximately the same elevation was carried out (Figure 14). However, two problems arose in this study. First, for some reason the elevation of the BeiDou satellites was not reported by the RTKLIB software, so one was chosen (C13) without actually knowing its elevation. Second, during the experiment, the only GLONASS satellite above 40 degrees was R21, which had much higher elevations (around 90 degrees) than the other two satellites chosen (E24 and G31). Bearing this in mind, it can be seen that the jammer did not affect GLONASS and BeiDou satellites, except for the region in very close proximity to the jammer. This can be probably attributed to their working frequency range that is slightly above the jammer spectrum. On the other hand, when comparing GPS and Galileo satellites that work in the same frequency band, it can be seen that the former had a more uniform dependency in the contrast to the latter that exhibited a significant signal drop in a region around 150 m.

### 3.3. Recovery from Jamming

**Figure 15:**
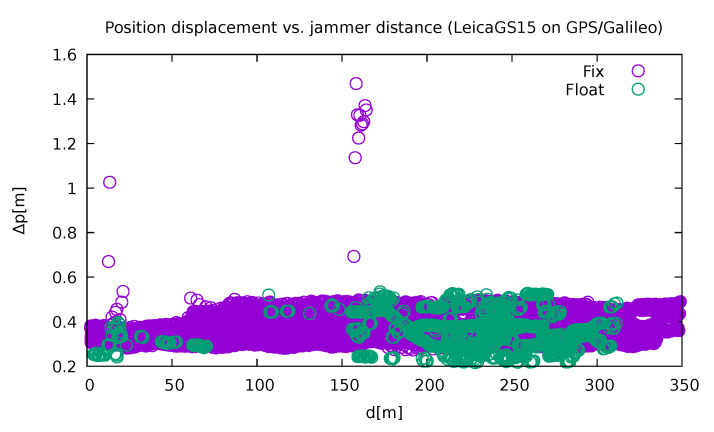
Position displacement for Leica GS15 using GPS and Galileo. Note a considerable discrepancy at a distance of 160 m from the jammer (own study, from the collection [43]).

In most of the cases, the instruments recovered quickly and correctly after being jammed. However, there was a notable exception in the case of Leica GS15 (Figure 15). There was a discrepancy at 160 m from jammer about 1.5 m from its actual position. Furthermore, the instrument reported those wrong solutions as fixed.

**Figure 16:**
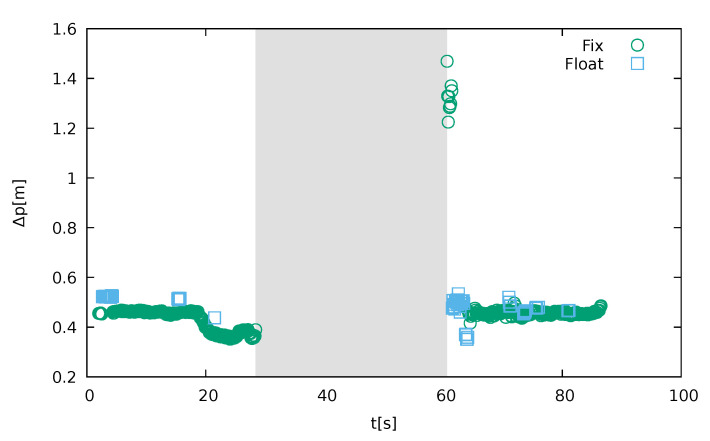
Time dependency of the position displacement for Leica GS15 using GPS and Galileo. For the sake of clarity, only the affected drive is shown. The shadowed region is 32.2 s wide and represents the region with no solutions at all (own study).

Such a discrepancy far from the jammer can be explained if the time dependency is inspected (Figure 16). For the sake of clarity, only the drive with the anomaly present is shown. This corresponds to drive 10 in Table 2. It is clearly visible there that the instrument lost its capability to give a proper solution for 32.2 s. This started when it was approximately 80 m ahead of the jammer. Then it did not recover until 190 m behind. Although both the distances are particularly long, the latter clearly indicates that the instrument went into a state of inability to recover from jamming. After it finally started reporting solutions, it gave a solution that differed by approximately 1 m from the real position, despite declaring it fixed. Clock biases also recovered after the receiver has passed the jammer, as shown in the Figure 9, but the deviations start already when the receiver is under the influence of the low power of the jammer, as observed by [1] in a controlled environment. Clock-bias variations decay after the receiver passes the jammer at a constant speed. The absence of any other sources in line-of-sight conditions suggests that the only reason for the significant clock-bias variations is intentional jamming.

## 4. Conclusions

Geodetic GNSS receivers are not intended for vehicle navigation, but can be used to assess the quality of low-cost receivers that will be massively deployed for navigation purposes, including autonomous driving and UAV direct georeferencing. The latest generation of such receivers can receive satellite signals from all constellations on several frequencies, making them comparable to a certain degree with professional geodetic receivers. In this research, a comparison between geodetic and low-cost receivers was estimated for kinematic positioning during static jamming, and their responses were analysed. The findings of this experiment led to the following conclusions:

- All of the receivers’ reported positions fluctuated around a few values that eventually changed during the experiment;
- The presented referencing method does not perform well in areas where the position is non-predictable (e.g., turns and non-uniform speed);
- The actual GLONASS constellation was not optimal, due to the lack of satellites of high elevation (above 40 degrees);
- From the observed CNR dependence, it can be safely assumed that none of the used instruments has any effective type of mitigation technique adapted to the chirp jammer.

All tests were performed under open sky GNSS conditions in the test area, where there were almost no obstacles that could be proven to be a source of satellite signal loss or multipath effects, except for a small fragment of the road near one of the turning points, where the present trees screened the signal from satellites. To gain more insight into evaluating the performance of GNSS receivers in kinematic mode, further testing should be conducted in the future to account for multipath effects due to the position of the receivers on the metal roof of the vehicle, which appears to be a source of reflections and multipath effects. In this study, multipath was estimated by the linear combination of Melbourne Wübbena for all receivers used in the experiments, but additional tests that consider different receiver environments (metal and non-metal) should be an issue to be resolved in the future. In this study, the focus was on jamming, and the investigation was based on the fact that all receivers were mounted on the same vehicle, which could be a source of unwanted reflections. In addition, positioning should also be improved by the use of inertial instruments, especially in less ideal environments where various factors, particularly fading and unwanted electromagnetic interference, can affect positioning performance.

## Figures and Tables

**Table 1:** Locations in the ETRS89 coordinate system of the most distinct points of driving, towards Petrinje (P) and Črnotiče (C). The height of the jammer (J) is related to the point on the ground, but the jammer was set at different points above the ground. Heights (H) were acquired from the ellipsoidal heights by using the SLOVRP2016/Koper geoid model.

Point Name	B-Latitude	L-Longitude	H [m]
Črnotiče (C)	45${}^{\circ }$33${}^{\prime }$40.078${}^{\prime \prime }$ N	13${}^{\circ }$53${}^{\prime }$29.889${}^{\prime \prime }$ E	439.90
jammer (J)	45${}^{\circ }$33${}^{\prime }$49.124${}^{\prime \prime }$ N	13${}^{\circ }$53${}^{\prime }$38.248${}^{\prime \prime }$ E	435.49
Petrinje (P)	45${}^{\circ }$34${}^{\prime }$01.064${}^{\prime \prime }$ N	13${}^{\circ }$53${}^{\prime }$48.894${}^{\prime \prime }$ E	433.91

**Table 2:** Vehicle speed, direction and times for each test drive.

No. of Drive	Vehicle Speed	Direction	Start (UTC)	End (UTC)
1	30 km/h	J–C	14:07:20	14:07:50
2	30 km/h	C–P	14:09:10	14:10:50
3	30 km/h	P–C	14:12:25	14:14:05
4	30 km/h	C–P	14:14:25	14:16:05
5	30 km/h	P–C	14:16:40	14:18:20
6	30 km/h	C–P	14:21:35	14:23:15
7	30 km/h	P–C	14:23:55	14:25:35
8	30 km/h	C–P	14:26:00	14:27:40
9	30 km/h	P–C	14:28:15	14:29:55
10	30 km/h	C–P	14:30:40	14:32:20
11	30 km/h	P–C	14:34:30	14:36:10
12	30 km/h	C–P	14:38:20	14:40:00
13	30 km/h	P–C	14:40:30	14:42:10
14	30 km/h	C–P	14:42:55	14:44:35
15	80 km/h	P–C	14:45:05	14:46:45
a short break			14:49:00	
16	60 km/h	P–C	14:51:00	14:51:55
17	60 km/h	C–P	14:52:40	14:53:20
18	60 km/h	P–C	14:54:20	14:55:05

**Table 3:** Locations in the ETRS89 coordinate system of three base receivers. Heights (H) were acquired from the ellipsoidal heights by using the SLOVRP2016/Koper geoid model.

Point Name	B-Latitude	L-Longitude	H [m]
B1	45${}^{\circ }$33${}^{\prime }$47.91960${}^{\prime \prime }$ N	13${}^{\circ }$53${}^{\prime }$46.79756${}^{\prime \prime }$ E	431.950
B2	45${}^{\circ }$33${}^{\prime }$54.67516${}^{\prime \prime }$ N	13${}^{\circ }$53${}^{\prime }$36.16875${}^{\prime \prime }$ E	433.722
B3	45${}^{\circ }$33${}^{\prime }$41.97677${}^{\prime \prime }$ N	13${}^{\circ }$53${}^{\prime }$30.83599${}^{\prime \prime }$ E	438.648

## Data Availability

The data that support the findings of this study are openly available at https://gnss.fpp.uni-lj.si/2021-03-19/ (accessed on 1 July 2021). Additional data can be available on request from the corresponding author.

## Abbreviations

The following abbreviations are used in this manuscript:

AKOS	Agency for Communication Networks and Services of the Republic of Slovenia
CORS	Continuously Operating Reference Station
C/N0	Carrier-to-Noise density
CNR	Carrier-to-Noise Ratio
GLONASS	Russian: Globalnaya Navigatsionnaya Sputnikovaya Sistema
GNSS	Global Navigation Satellite Systems
GPS	Global Positioning System
RINEX	Receiver Independent Exchange Format
RIMS	Ranging and Integrity Monitoring Station
RTK	Real Time Kinematic
UTC	Coordinated Universal Time
SIGNAL	SI-Geodesy-Navigation-And-Location
STRIKE3	Standardisation of GNSS Threat reporting and Receiver testing through
	International Knowledge Exchange, Experimentation and Exploitation
